# Supplementary material for: Transfer printing micro-assembly of silicon photonic crystal cavity arrays: beating the fabrication tolerance limit
Source: Nat Commun. 2025 Jul 1;16:5994. doi: 10.1038/s41467-025-60957-1 (PMC12217402; doi:10.1038/s41467-025-60957-1)
Supplement: Supplementary file 1 — Supplementary Information [file 41467_2025_60957_MOESM1_ESM.pdf]

Transfer printing micro-assembly of silicon photonic crystal cavity arrays: beating the fabrication tolerance limit

Sean P. Bommer[1*], Christopher Panuski[2], Benoit Guilhabert[1], Zhongyi Xia[1], Jack A. Smith[1], Martin D. Dawson[1], Dirk Englund[2], Michael J. Strain[1*]

[1]Institute of Photonics, Dept. of Physics, University of Strathclyde, UK.
[2]Research Laboratory of Electronics, MIT, USA.

*Corresponding author(s). E-mail(s): sean.bommer@strath.ac.uk; michael.strain@strath.ac.uk;

1 Transfer printing of photonic crystal cavities

1.1 Printing process

The general method used to transfer print objects using a polymeric stamp is well covered in the literature [1–4] and typically relies on the reversible adhesion of the stamp-object interface based on competitive adhesion effects between the stamp and the donor and receiver substrates, making use of velocity control during the contact phases of the process [5]. A further variation on this basic method has been developed to reduce surface contact areas during the print phase using protrusions that can dynamically relax [6]. These methods are suitable for membranes with thicknesses greater than approximately 1 μm or with high stiffness, but the flexibility of thin device membranes, or micron-scale devices, require the use of flat stamp surfaces to avoid unwanted deformation of the devices [7]. Furthermore, the composition of the polymeric material can be engineered to control its surface adhesion to promote transfer in the required direction from donor to receiver [7].

In this work, the small-scale of the membranes and the sub-micron thickness necessitated use of a flat stamp surface, using a Polydimethylsiloxane (PDMS) composition

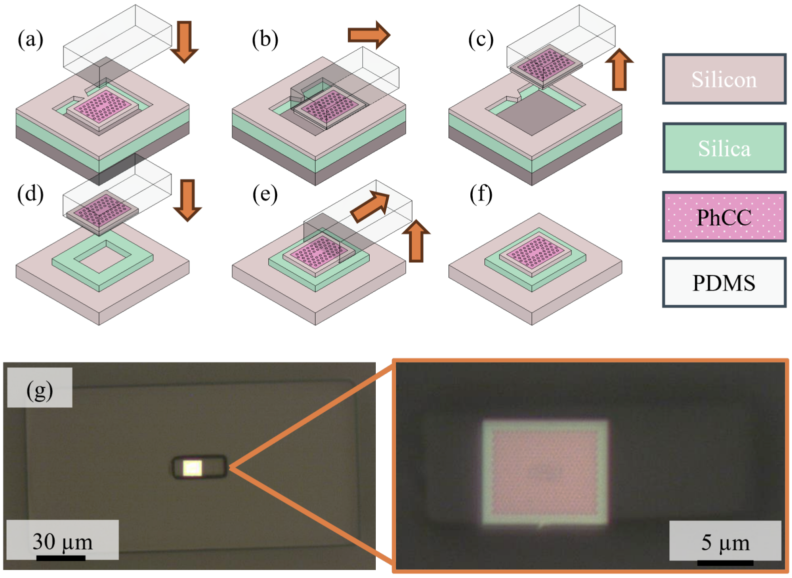

Supplementary Fig. 1 μ **-transfer print process for suspended photonic crystal cavities.** (a) The stamp is aligned to the donor device. (b) A lateral motion at a velocity of $8000\mu m/s$ is used to break the device anchor. (c) The device is removed from the donor chip. (d) The picked device is aligned to a silica frame on the receiver chip and brought into contact. (e) Device is released onto the receiver frame using a combined sheer and vertical motion at a velocity of $0.05\mu ms^{-1}$. (f) The stamp is removed from the device to ensure device is printed. (g) Microscope view of transfer print stamp with printed device.

of 10:1 (monomer:curing agent) mixture. A schematic of the transfer process is presented in Supplementary Fig. 1, along with images of a PhCC attached to the polymer stamp head.

The PhCC's are lithographically defined with a single, laterally tapered tether with a minimum dimension of $2\,\mu m$ at the membrane edge. This tether was designed to fracture at the minimum cross-section and all 249 devices (including the ordered wavelength device set, the repeat print cycle set and the dense integration set) transferred successfully with the tether cleaving at the designed point with no detectable physical damage to the PhCC pixel. The flat stamp surface was brought into contact with the PhCC surface, ensuring no stamp contact with the tether area. Once in contact a lateral shear motion of $\approx 1\,\mu m$ was applied to fracture the tether at a velocity of $\approx 8000\,\mu ms^{-1}$, Supplementary Fig. 1(a). The PhCC on the stamp is then aligned to the receiver chip suspension frame, centring the PhCC on the air void at the centre of the frame. The PhCC is brought into contact with the silica suspension frame, then released from the stamp surface using a combined shear and vertical motion at a velocity of $\approx 0.05\mu ms^{-1}$. This releases the PhCC into its target position with a yield of 100% for the 448 prints carried out for this work, i.e. 238 prints of the main cavity set, 90 prints of the repeatability tests, and 120 prints for the dense integration set.

1.2 Contact area measurements

To quantify the extent of the spatial overlap between the printed PhCC devices and support frames, a frame was imaged in a scanning electron microscope before and after printing of the PhCC, as shown in Supplementary Fig. 1.2. The internal dimensions of the support frame were measured as $8.5 \times 7.5\,\mu m$ prior to the PhCC printing. The PhCC device was measured with external dimensions of $11 \times 9\,\mu m$, and by image overlap analysis of the pre- and post-printing images the mean overlap lengths between PhCC and support frame were $1.21, 1.24, 0.49, 1.05\,\mu m$ for the left, right, top and bottom edges respectively resulting in an average overlap of $1.00\mu m$. The edge detection was performed within Python using standard techniques within the OpenCV package, using the following process. A Gaussian blur was used to de-noise the image, following a morphological close operation to remove pixel islands that were unattached to the target structures. Finally, an adaptive threshold was used to binarize the image. An averaged line profile was taken for each edge of the imaged structures, in both the horizontal and vertical directions and passed through a peak detection algorithm from the SciPy library to find the locations of each edge, and the errors are taken as the FWHM of each imaged structure edge.

2 In-situ optical measurement system

2.1 Optical system

A schematic of the in-situ optical measurement system is shown in Supplementary Fig. 3. The swept tuneable laser source, Agilent 8614B, is coupled through polarisation maintaining fibres to a parabolic fibre to free space collimating mirror. The collimator is directly coupled to a scanning galvonometer and lens system for beam steering at the sample plane. The laser source has a linear polarisation with $> 20\,$dB extinction and is aligned at a 45° angle to the x-axis of the translation stage and the mounted samples. The polarisation filter in the reflected beam path is set at an angle of 90° with respect to the input polarisation to reject specular reflection from the sample surface and microscope column optics. The polarisation of the PhCC mode is then at 45° with respect to both the input polarisation and output polarisation angle, inducing a 6dB loss in throughput, but suppressing the specular reflection background. The reflected signal is then focussed on an InGaAs focal plane array for detection. The InGaAs array is housed inside an Lucid Vision Triton Global Shutter camera, the bandgap of the sensor has been optimised to extend the sensitivity down to visible wavelengths, facilitating a broad range of $400 - 1700\,$nm. This allows co-alignment of wide field illuminated device structures to the injected tuneable IR beam using the same sensor.

2.2 Spectral measurement method

The spectral measurements of PhCCs were based on swept tuneable laser sampling. The tuneable laser source was an Agilent 8164A system, running in continuous sweep mode over a 10 nm wavelength range around 1550 nm at a constant sweep speed of 0.5 nm/s. The reflected optical signal from the cavity was measured on the InGaAs focal array, imaging at a maximum frame rate of 833.3 fps. The camera integration

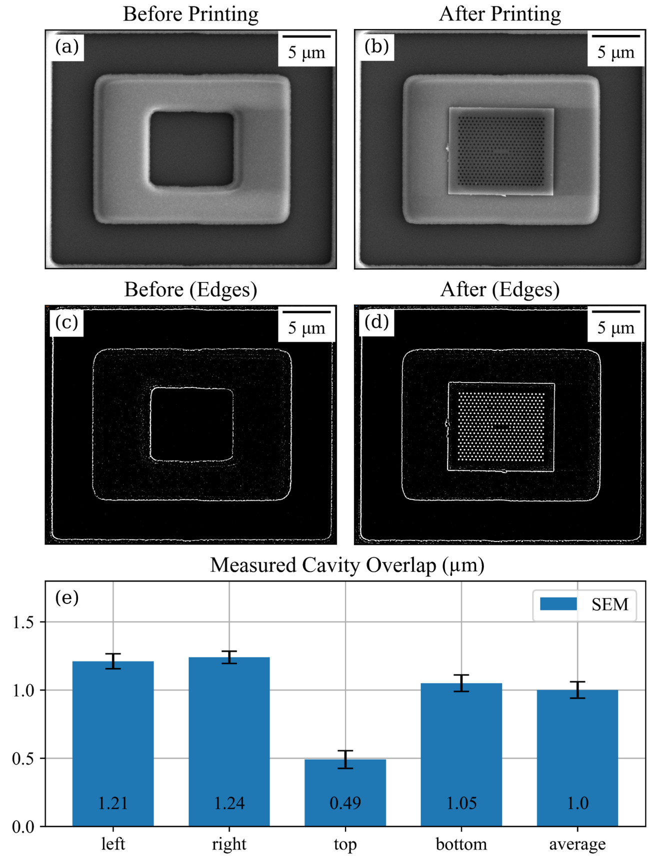

Supplementary Fig. 2 SEM analysis of Cavity Overlap (a-b) SEM images of silica support frame before and after printing of PhCC device. (c-d) Post edge detection process for before and after printing. (e) Measured overlap of each cavity edge, error bars represents the combined FWHMs of the average line profile of (c) and (d).

time was set by the frame rate and the gain was adjusted to avoid pixel saturation at the maximum reflected signal level. To calculate the reflection intensity in a single camera frame sample period, a region of interest was defined in the image, which remained constant across all frames, and a summation carried out across the pixels in that area. Representative scans of sample PhCCs in an array were carried out to avoid image saturation, whilst maintaining effective use of the camera's dynamic range. The signal from each frame was then translated through time sampling to a corresponding laser wavelength. Given a laser sweep speed of $0.5nm/s$ and an imaging frame rate of 833.3 fps, the measurement spectral resolution was 0.6 pm. This could be improved for higher Q-factor cavity systems by using a slower laser sweep rate. For dynamic measurements the laser scan range was reduced to 2.5 nm to enable faster measurements.

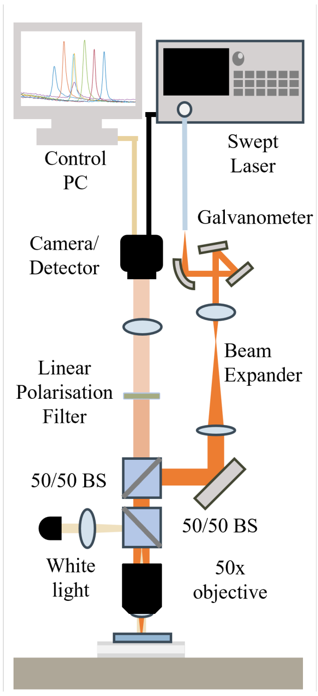

Supplementary Fig. 3 Schematic of the optical measurement system. The system comprises tuneable laser source and broadband white light illumination, galvo beam steering, microscope column, polarisation filter, and camera detection system.

2.3 Extraction of resonant wavelength and cavity Q-factor

The resonance wavelength and linewidth were evaluated by fitting the measured reflectivity spectra data around a resonance to an analytical model. Following common practice in literature, we have used a Fano resonance model for fitting our photonic crystal cavity spectra [8–11]. After removing the low frequency background modulation from the signal, we fit the reflected intensity, $I(\lambda)$, with the Fano equation [11] shown in Eq.[1]. The fitting was carried out using a nonlinear regression method in MATLAB.

$$I(\lambda) = A + B\frac{(cot(\delta) + 2(\lambda - \lambda_R)/\gamma)^2}{1 + (2(\lambda - \lambda_R)/\gamma)^2} \tag{1}$$

Here, λ_R is the resonant wavelength, γ is the resonance width, and A and B are constants relating to the background and peak height respectively. The parameter controlling the deviation from Lorentzian lineshape is δ, which refers to the phase shift from the coupling of a discrete resonant mode to a continuum band of states [11]. It is related to the Fano parameter, q, through $q = cot\delta$, and it can be shown that for $q \to 0$ or $q \to \pm\infty$ Lorentzian lineshapes are recovered. The Q factors are measured as $Q = \lambda_R/\gamma$.

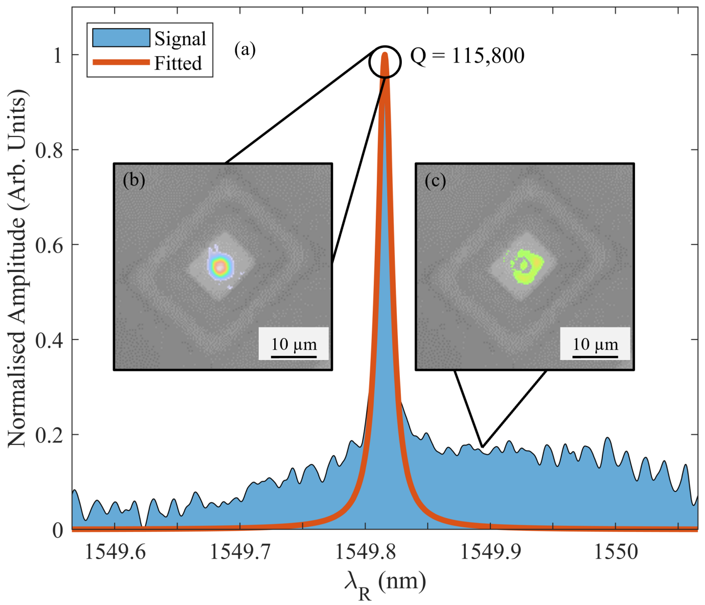

Supplementary Fig. 4 Spectral measurement of PhCC in the transfer system. (a) Measured reflectivity spectrum from a PhCC captured using the in-situ measurement system. The PhCC is printed on a receiver substrate silica suspension frame. Insets show the spatial mode images captured by the InGaAs camera at two points in the tuneable wavelength sweep corresponding to on-resonance (b) and off-resonance (c) conditions.

2.4 Cavity Q-factors effects

In addition to the main results presented in the manuscript that relate to the cavity resonant wavelength, cavity Q-factors are another important element in the transferred device performance. The Q-factor was calculated as detailed above for the set of 10 cavities that were printed over 9 successive cycles, corresponding to Figure 5 in the main text. Supplementary Fig. 5 shows the measured Q-factor of the cavities for each print cycle along with the relative difference in Q-factor for devices between cycles. The average Q-factor is in the 10^5 range, and although individual device variations between print cycles can be in the 10^4 range, the average value is relatively stable over the full set of print cycles. So although Q-factor is more susceptible to variation than the cavity resonant wavelength, devices remain within a consistent operation range, allowing targeting with resonant wavelength as primary characteristic.

3 Spatial clusters of cavity resonances within ordered arrays

The ordered arrays of devices maintained the overall standard deviation of resonant wavelength, λ_R, from the as-fabricated PhCC devices. The ordering allows for local spatial clusters of devices with closely spaced resonant wavelengths to be assessed. The average cavity linewidth of ~ 0.015 nm was taken as a first approximation of the maximum deviation between individual cavity resonant wavelengths that would allow

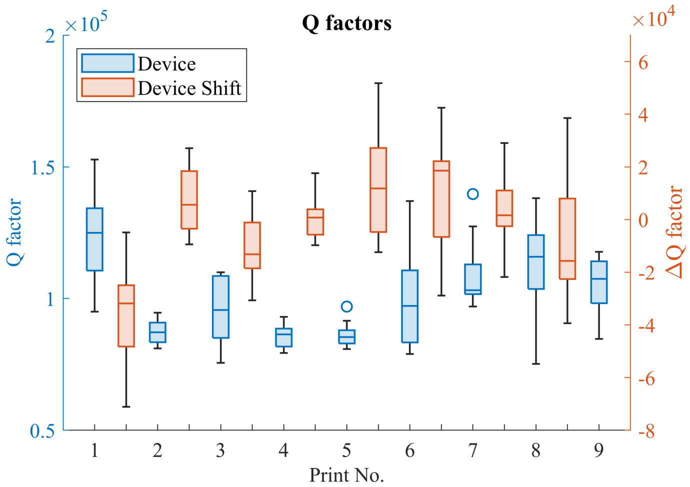

Supplementary Fig. 5 Q-factor variation as a function of repeated print cycle Q-factor variation as a function of repeated print cycle for a set of 10 PhCC devices. Absolute Q-factor value for the 10 device set is presented along with the device specific Q-factor variation associated with each print cycle. Box plots indicate median value (middle line), 25th, 75th percentile (box) and 5th and 95th percentile (whiskers) as well as outliers (single points).

them to interact with a single narrow linewidth laser source. Supplementary Fig. 6 (a) shows the distribution of PhCC clusters across the range of measured resonant wavelengths. The bubble size is related to the number of devices with resonant wavelengths within the average cavity linewidth from one another. There are a number of groupings on the order of 5-6 cavities, showing that even without post-fabrication tuning device selection and spatial binning can be achieved even within a set of only 119 devices. Supplementary Fig.6(b-d) shows examples of cavity resonance clusters around a wavelength of 1550nm.

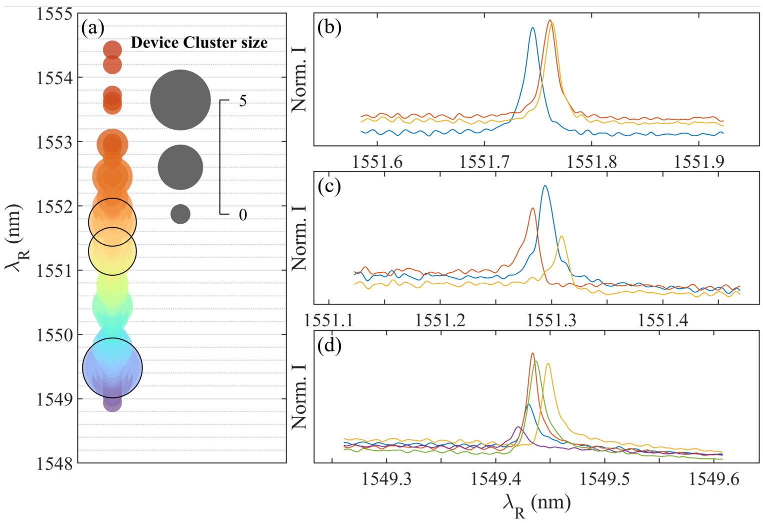

Supplementary Fig. 6 Cavity Clustering. (a) A bubble chart showing resonant wavelength ordered on the y-axis, spatial position by colour and number of devices within a standard deviation of 0.015 nm by bubble size. (b-d) Selected groupings of devices outlined in black in (a) are shown as reflectivity spectra to show the spectral overlap between devices. axes are of normalised intensity of measured on the InGaAs camera versus wavelength.

References

[1] Carlson, A. *et al.* Active, programmable elastomeric surfaces with tunable adhesion for deterministic assembly by transfer printing. *Advanced Functional Materials* **22**, 4476–4484 (2012).

[2] Carlson, A., Bowen, A. M., Huang, Y., Nuzzo, R. G. & Rogers, J. A. Transfer printing techniques for materials assembly and micro/nanodevice fabrication. *Advanced Materials* **24**, 5284–5318 (2012).

[3] Corbett, B., Loi, R., Zhou, W., Liu, D. & Ma, Z. Transfer print techniques for heterogeneous integration of photonic components. *Progress in Quantum Electronics* **52**, 1–17 (2017).

[4] Smith, J. A., Jevtics, D., Guilhabert, B., Dawson, M. D. & Strain, M. J. Hybrid integration of chipscale photonic devices using accurate transfer printing methods. *Applied Physics Reviews* **9**, 041317 (2022).

[5] Feng, X. *et al.* Competing fracture in kinetically controlled transfer printing. *Langmuir* **23**, 12555–12560 (2007).

[6] Kim, S. *et al.* Microstructured elastomeric surfaces with reversible adhesion and examples of their use in deterministic assembly by transfer printing. *Proceedings of the National Academy of Sciences of the United States of America* **107**, 17095–17100 (2010).

[7] Jevtics, D. *et al.* Spatially dense integration of micron-scale devices from multiple materials on a single chip via transfer-printing. *Optical Materials Express* **11**, 3567 (2021).

[8] Galli, M. *et al.* Light scattering and fano resonances in high-q photonic crystal nanocavities. *Applied Physics Letters* **94** (2009).

[9] Wu, C. *et al.* Spectrally selective chiral silicon metasurfaces based on infrared fano resonances. *Nature communications* **5**, 3892 (2014).

[10] Yang, Y., Kravchenko, I. I., Briggs, D. P. & Valentine, J. All-dielectric metasurface analogue of electromagnetically induced transparency. *Nature communications* **5**, 5753 (2014).

[11] Limonov, M. F., Rybin, M. V., Poddubny, A. N. & Kivshar, Y. S. Fano resonances in photonics. *Nature photonics* **11**, 543–554 (2017).
